# Membrane Transporters for Sulfated Steroids in the Human Testis - Cellular Localization, Expression Pattern and Functional Analysis

**DOI:** 10.1371/journal.pone.0062638

**Published:** 2013-05-08

**Authors:** Daniela Fietz, Katharina Bakhaus, Britta Wapelhorst, Gary Grosser, Sabine Günther, Jörg Alber, Barbara Döring, Sabine Kliesch, Wolfgang Weidner, Christina E. Galuska, Michaela F. Hartmann, Stefan A. Wudy, Martin Bergmann, Joachim Geyer

**Affiliations:** 1 Institute for Veterinary Anatomy, Histology and Embryology, Justus Liebig University Giessen, Giessen, Germany; 2 Institute for Veterinary Pharmacology and Toxicology, Justus Liebig University Giessen, Giessen, Germany; 3 Department of Clinical Andrology, Centre for Reproductive Medicine and Andrology, University Hospital Münster, Münster, Germany; 4 Clinic for Urology, Pediatric Urology and Andrology, Justus Liebig University Giessen, Giessen, Germany; 5 Steroid Research and Mass Spectrometry Unit, Division of Paediatric Endocrinology and Diabetology, Center of Child and Adolescent Medicine, Justus Liebig University Giessen, Giessen, Germany; John Hopkins University School of Medicine, United States of America

## Abstract

Sulfated steroid hormones are commonly considered to be biologically inactive metabolites, but may be reactivated by the steroid sulfatase into biologically active free steroids, thereby having regulatory function via nuclear androgen and estrogen receptors which are widespread in the testis. However, a prerequisite for this mode of action would be a carrier-mediated import of the hydrophilic steroid sulfate molecules into specific target cells in reproductive tissues such as the testis. In the present study we detected predominant expression of the Sodium-dependent Organic Anion Transporter (SOAT), the Organic Anion Transporting Polypeptide 6A1, and the Organic Solute Carrier Partner 1 in human testis biopsies. All of these showed significantly lower or even absent mRNA expression in severe disorders of spermatogenesis (arrest at the level of spermatocytes or spermatogonia, Sertoli cell only syndrome). Only SOAT was significantly lower expressed in biopsies showing hypospermatogenesis. By use of immunohistochemistry SOAT was localized to germ cells at various stages in human testis biopsies showing normal spermatogenesis. SOAT immunoreactivity was detected in zygotene primary spermatocytes of stage V, pachytene spermatocytes of all stages (I–V), secondary spermatocytes of stage VI, and round spermatids (step 1 and step 2) in stages I and II. Furthermore, SOAT transport function for steroid sulfates was analyzed with a novel liquid chromatography tandem mass spectrometry procedure capable of profiling steroid sulfate molecules from cell lysates. With this technique, the cellular inward-directed SOAT transport was verified for the established substrates dehydroepiandrosterone sulfate and estrone-3-sulfate. Additionally, β-estradiol-3-sulfate and androstenediol-3-sulfate were identified as novel SOAT substrates.

## Introduction

Sulfated steroid hormones for long have been merely regarded as biologically inactive steroid metabolites. However, increasing evidence came up during the last decades that hydrolysis of steroid sulfates catalyzed by the steroid sulfatase (StS) is an important alternative source of precursors for the local supply of estrogens and androgens via the so-called sulfatase pathway [1], [2]. In humans, StS has been identified as a valuable drug target for estrogen and androgen deprivation therapies in hormonal diseases [3]. Thus, in addition to the provision of steroid hormones by the secretory activity of a given cell or gland, a second system controlling the availability of biologically active steroids on the cellular level might be established due to the expression of StS and/or estrogen sulfotransferases in certain organs, like the testis [4], [5].

Across species, Leydig cells in adult testes are the primary source of testicular androgens and also estrogens via *de novo* biosynthesis [6]. Besides the free steroid forms, the human testis is also able to produce steroid sulfates including pregnenolone sulfate (PREGS), dehydroepiandrosterone sulfate (DHEAS) and testosterone sulfate [7], [8], [9]. The primary targets of the androgens in the testis are Leydig cells, Sertoli cells and peritubular cells, due to their expression of androgen receptor (AR) [10]. Furthermore, two isoforms of the estrogen receptor (ERα and ERβ) are expressed in the efferent duct epithelium and showed importance for fluid re-absorption [6], [11], [12]. Expression of ERα and ERβ mRNA and protein was also demonstrated in spermatogonia, primary spermatocytes (ERα) and Sertoli cells (ERβ) in the boar [13], as well as ERα mRNA in spermatogonia and primary spermatocytes in human, dog, mouse and horse [14], for review see [15]. Therefore, the testis is responsive not only to androgens, but also to estrogens.

In addition to free steroid hormones, the sulfated forms, like PREGS and DHEAS, may have regulatory function in the testis as they can be used as precursors for testosterone production [16], [17], [18], [19]. As it was shown that PREGS cleavage by StS in the human testis is principally concentrated in seminiferous tubules rather than in interstitial tissue, testosterone may be synthesized in the direct surrounding of germ cells [17]. Interestingly, in patients showing StS deficiency (recessive X-linked ichthyosis), a high proportion of males show associated testicular diseases including cryptorchidism and elevated steroid sulfate levels [20], [21]. This indicates that the recruitment of sulfated steroids over the sulfatase pathway might contribute to the maintenance of normal spermatogenesis in men [1].

Although the synthesis and cleavage of sulfated steroids was investigated in the testis, the question of how the negatively charged sulfated steroid hormones can pass through the cell membrane of specific target cells in the testis has not been answered. However, several membrane carriers exist in the testis that show transport activity for different kinds of anionic organic molecules including steroid sulfates [22], [23], [24]. Most of them, e.g. the members of the Organic Anion Transporting Polypeptide (OATP) family (Solute Carrier Family SLCO), have broad and overlapping substrate specificities [25], [26]. In contrast, the Sodium-dependent Organic Anion Transporter SOAT (Solute Carrier Family SLC10, member SLC10A6), identified by our group in 2004 [27], seems to be specific for the transport of steroid sulfates [28], [29]. SOAT was shown to be highly expressed in the human testis and, therefore, is a candidate carrier for the local supply of steroid sulfates in the testis. Besides SOAT, OATP6A1 (SLCO6A1) and OATP1C1 (SLCO1C1) as well as the Organic Solute Carrier Partner 1 (OSCP1) showed high expression in the testis. Only for mouse Oscp1 the cellular localization in the testis has been analyzed more closely to date and revealed differing expression in leptotene spermatocytes at stage IX onwards until step 15 spermatids [30], [31] or in the plasma membrane of Sertoli cells [32].

The aim of our study was to investigate the expression pattern and cellular localization of SOAT and other membrane carriers in the human testis and to quantify their expression in patients with impaired spermatogenesis. Furthermore, we aimed to identify the most relevant carrier for steroid sulfates in the testis and to verify inward-directed transport of the entire steroid sulfate molecule with liquid chromatography tandem mass spectrometry (LC-MS-MS) procedure that is capable of profiling intact steroid sulfates from cell lysates.

## Materials and Methods

### Ethics Statement

All testicular biopsies were taken after written informed consent under general anesthesia at the Department for Clinical Andrology, Center for Reproductive Medicine and Andrology at the University of Münster or at the Department of Urology at University Clinics Giessen-Marburg (UKGM) of Giessen. The reported study has been approved by the Ethics committee of the Medical Faculty of the Justus Liebig University Giessen (decision 187b/09). Cultured Sertoli cells (FS1) are derived from testis tissue of an adult man with Fraser syndrome (FS) and were obtained at the time of prophylactic gonadectomy after written informed consent [33].

### Testicular Tissue and Histological Evaluation

We evaluated 44 testis biopsies, indicated because of normo- or hypergonadotropic azoospermia (for review see [34]) including patients with obstructive azoospermia after vasectomy. After surgery, testicular tissue was fixed by immersion in Bouin’s solution and embedded in paraffin. For histological evaluation, 5 µm thick sections were cut, stained with hematoxylin and eosin and evaluated following score count analysis according to Bergmann and Kliesch [34]. For our study, testicular biopsies revealing normal spermatogenesis (nsp, n = 12), qualitatively intact but quantitatively reduced spermatogenesis (hypospermatogenesis, hyp, n = 5), arrest at the level of primary spermatocytes (sza, n = 13) or spermatogonia (sga, n = 5) as well as Sertoli cell only syndrome (SCO, n = 9) were used. Additionally, immortalized and cultured human Sertoli cells (FS1, kindly provided by Dr. V. Schumacher, Children’s Hospital Boston, [33]) were used for quantitative RT-PCR experiments. Cells were cultured in DMEM high glucose (4.5 g/L, LifeTechnologies, Carlsbad, CA, USA), 20% fetal calf serum (FCS Gold, PAA, Pasching, Austria), 1% non-essential amino acids, 1% L-glutamine and 1% penicillin-streptomycin (LifeTechnologies). Incubation was conducted at a humidified atmosphere with 8.5% CO_2_ and a temperature of 37°C.

### Expression Pattern of SOAT, OATP6A1, OATP1C1 and OSCP1

Expression patterns of SOAT, OATP6A1, OATP1C1 and OSCP1 were examined by using human multiple tissue cDNA panels (BioChain, Newark, CA, USA). PCR amplification was achieved with TaqMan Gene Expression Assays (LifeTechnologies) for human SOAT, OATP6A1, OATP1C1, and OSCP1. Human β-actin and RNA polymerase II were used as endogenous controls (Table 1). For each specimen, triplicate determinations were performed using 5 µl of cDNA, 1.25 µl of the respective TaqMan Gene Expression Assay, 12.5 µl TaqMan Gene Expression Master Mix (LifeTechnologies) and aqua bidest to a final volume of 25 µl. Quantitative PCR was performed on an ABI Prism 7300 thermal cycler (Applied Biosystems, Darmstadt, Germany). Conditions were 1×95°C for 10 min and 45×(95°C for 15 s and 60°C for 1 min). Relative gene expression was calculated by the 2^−ΔΔCT^ method. Expression levels represent x-fold higher expression in the given tissue than in the tissue with the overall lowest expression level (set as a calibrator).

**Table 1:** TaqMan Gene Expression Assays used for quantitative real-time RT-PCR amplification.

Target	RefSeq	TaqMan Gene Expression Assay ID	amplicon (bp)
	(NCBI database)		
OATP6A1	NM_173488.3	Hs00542846_m1	63
OATP1C1	NM_017435.4	Hs00213714_m1	92
OSCP1	NM_145047.4	Hs00376771_m1	115
SOAT	NM_197965.2	Hs01399354_m1	119
β-actin	NM_001101.3	Hs99999903_m1	171
		Hs00357333_g1	77
GAPDH	NM_002046.3	Hs02758991_g1	93
RNA Pol II	NM_000937.4	Hs01108291_m1	86

### Quantitative Real-time PCR from Testicular Biopsies

For quantitative analysis of SOAT expression in testis homogenates, quantitative real-time PCR amplification was achieved with TaqMan Gene Expression assays. Human β-actin and glyceraldehyde 3-phosphate dehydrogenase (GAPDH) were used as endogenous control (Table 1). For each specimen, triplicate determinations were performed using 3 µl of cDNA, 1 µl of the respective TaqMan Gene Expression Assay, 10 µl TaqMan Gene Expression Master Mix (LifeTechnologies) and aqua bidest to a final volume of 20 µl. Amplification was performed as outlined above. For quantitative analysis of OATP6A1 and OSCP1 expression, PCR amplification was achieved with the same primers as used for qualitative RT-PCR (Table 2). Detection was performed by SYBR Green and subsequent melting curve analysis to ensure specificity of PCR products. For each specimen, triplicate determinations were performed using 1 µl of cDNA, 10 µl of iQ SYBR Green Supermix (Bio-Rad Laboratories, Hercules, CA, USA), 0.6 µl of each primer and aqua bidest to a final volume of 20 µl. Quantitative real-time PCR was performed on CFX96 Real-Time Cycler (Bio-Rad). For OATP6A1 and OSCP1, conditions were 1×95°C for 3 min and 40×(95°C for 10 s, 60°C for 1 min) and melting curve analysis (1×95°C for 10 s, 65°C to 95°C, increment 0.5°C for 5 s). Relative gene expression was calculated by the 2^−ΔΔCT^ method. Expression levels represent x-fold higher expression in the given sample than in the specimen with the overall lowest expression level (set as a calibrator). For statistical analysis, ANOVA was performed followed by a student’s t-test (SPSS 19.0, IBM, Munich, Germany).

**Table 2:** Primer sequences for qualitative RT-PCR.

Target	RefSeq	Primer sequence (5′ → 3′)	amplicon (bp)
	(NCBI database)		
SOAT	NM_197965.2	ACCTGGTCCTGGAGTCTTC *for*	79
		GAATGGTCAGGCACACAAG *rev*	
SOAT ISH		CTGCTGGCACTTTTTACCC *for*	330
		GGCACCTTCTTCATTCACC *rev*	
OATP6A1	NM_173488.3	CTGACAAACTGCGTTCTCTG *for*	77
		TTGATGGTCCAGGAATAGTCC *rev*	
OSCP1	NM_145047.4	CATGTACAGCGTGAATCAGC *for*	96
		AAGAGGGTTTGGAGCAATG *rev*	
GAPDH	NM_002046.3	CCAGGTGGTCTCCTCTGACTTC *for*	81
		GTGGTCGTTGAGGGCAATG *rev*	
ß-actin	NM_001101.3	GCGAGAAGATGACCCAGATC *for*	84
		CGTACAGGGATAGCACAGC *rev*	

### Carrier Expression in Testis Biopsies by Qualitative RT-PCR

Total mRNA from testis homogenate was extracted from Bouin fixed, paraffin embedded tissue using the RNeasy Micro FFPE Kit (Qiagen, Hilden, Germany) as recommended by the manufacturer. Subsequently, mRNA was incubated with RNase-free DNase I (10 U/L; Roche, Mannheim, Germany) and RNase Inhibitor (40 U/L; LifeTechnologies) to digest genomic DNA. cDNA was synthesized from 1.5 µl total mRNA by using 8.5 µl of RT-mix (GeneAmp Gold RNA PCR Core Kit, LifeTechnologies). Negative controls were performed by omitting the reverse transcriptase (RT) reaction. For RT-PCR, 5 µl of cDNA was added to 2 µl MgCl_2_, 4 µl 10×PCR Gold Buffer, 0.25 µl GOLDAmplitaq (Life Technologies), 1 µl of each primer (10 µmol/L) and aqua bidest to a final volume of 25 µl. RT-PCR was performed by using specific primers for SOAT, OATP6A1, and OSCP1. Beta-actin primers were used for control of cDNA quality. All oligonucleotide primers were obtained from Eurofins MWG Operon (Ebersberg, Germany) and are listed in Table 2. RT-PCR conditions were 1×95°C for 5 min, 40×(95°C for 30 s, 57°C for 30 s and 72°C for 30 s) and 72°C for 7 min for SOAT amplification as well as 1×95°C for 5 min, 40×(95°C for 30 s, 60°C for 30 s and 72°C for 30 s) and 72°C for 7 min for OATP6A1 and OSCP1 amplification. PCR products were separated by 2% agarose gel electrophoresis and visualized by SYBR Green I staining (Sigma-Aldrich, St. Louis, MO, USA). For an isolated examination of seminiferous epithelia and interstitial cells, we performed laser-assisted cell picking (LACP) using paraffin-embedded tissue. Slices mounted on PALM membrane slides (MembranSlide 0.1 PEN, Zeiss, Oberkochen, Germany) were stained with hematoxylin. The tissue of interest was excised and catapulted by PALM MicroBeam system and PALM Robo Software (Zeiss, Oberkochen, Germany). Specimens of seminiferous tubules and interstitial tissue (consisting predominantly of Leydig cells, blood vessels and connective tissue) were picked and stored separately. Extraction of mRNA was performed using RNeasy FFPE Kit (Qiagen). Digestion of genomic DNA, first strand cDNA synthesis and RT-PCR were performed as outlined above.

### Cellular Localization of SOAT mRNA by *in situ* Hybridization (ISH)

Digoxigenin (DIG)-labeled cRNA probes were generated as described previously [35]. Briefly, a 330 bp PCR product of SOAT was sub-cloned into the pCRII TOPO vector (LifeTechnologies) as recommended by the manufacturer. Primer pairs for PCR reaction were obtained from Eurofins MWG Operon (Table 2). The plasmid was transformed into One Shot Chemically Competent *E. coli* (LifeTechnologies), purified and sequenced by SRD (Scientific Research and Development, Bad Homburg, Germany). For the synthesis of cRNA probes, the plasmid was digested by using restriction enzymes *Not* I or *BamH* I (NEB, Schwalbach, Germany). Subsequently, *in vitro* transcription of plasmid DNA in cRNA was performed using 10×RNA-DIG Labeling Mix (Boehringer Mannheim, Mannheim, Germany) and RNA polymerases T7 and SP6 (Promega, Heidelberg, Germany). ISH was performed as described by Lekhkota et al. 2006 [13] with minor changes. Deparaffinized and rehydrated testis sections (5 µm) were digested using 15 µg/ml proteinase K in 1×phosphate buffered saline (PBS, 137 mM NaCl, 2.7 mM KCl, 1.5 mM KH_2_PO_4_, 7.3 mM Na_2_HPO_4_) for 30 min at 37°C, post-fixed with 4% paraformaldehyde for 10 min, exposed to 20% acetic acid, and prehybridized in 20% glycerol for 1 h at room temperature. Afterwards, sections were incubated with the DIG-labeled sense or antisense cRNA probe. Both cRNAs were used at a dilution of 1∶50 in hybridization buffer containing 50% deionized formamide, 10% dextran sulfate, 2× standard saline citrate (SSC), 1× Denhardt's solution, 10 µg/ml salmon sperm DNA, and 10 µg/ml yeast t-RNA (Sigma-Aldrich). Hybridization was performed overnight at 37°C in a humidified chamber containing 50% formamide in 2×SSC. Post-hybridization washes were performed in 4×SSC at 37°C. Subsequently, sections were incubated with an anti-DIG Fab antibody conjugated to alkaline phosphatase (Boehringer) overnight at 4°C. Staining was visualized by developing sections with NBT-BCIP solution in a humidified chamber protected from light. Finally, sections were mounted with Kaiser’s glycerol gelatine (Merck, Darmstadt, Germany).

### Detection of SOAT in the Testis by Immunohistochemistry and Immunofluorescence

Although several antibodies are commercially available for human SOAT, none were applicable for immunohistochemistry or Western blot analysis. Therefore, we generated two antibodies, the first one against the whole C-terminus of the human SOAT protein (SOAT_311–377_ antibody) and the second one against the 16 C-terminal amino acids of the mouse Soat protein (Soat_329–344_ antibody). Rabbits were immunized with these peptides by Eurogentec (Liège, Belgium). The rabbit antisera were affinity-purified against the immunizing peptides and immunoreactivity was verified by enzyme linked immunosorbent assay. Then, both antibodies were used for immunohistochemistry (IHC), immunofluorescence (IF) and Western blot (WB). For IHC, paraffin sections from four testes with normal as well as from six testes with impaired spermatogenesis were deparaffinized and rehydrated. Heat mediated antigen retrieval was performed in citrate buffer solution (pH 6) for 15 min in a common microwave oven. For inhibition of endogenous peroxidase activity, sections were incubated in 3% H_2_O_2_ solution in Tris buffer for 30 min. Subsequently, sections were treated with 5% bovine serum albumin (BSA) for 30 min and the Soat_329–344_ antibody (dilution 1∶20) was incubated at 4°C overnight. The biotinylated goat anti-rabbit E0432 antibody (Dako, Glostrup, Denmark, dilution 1∶200) served as secondary antibody and was incubated at room temperature for 30 min. Immunoreactivity was visualized by peroxidase conjugated streptavidin (Vectastain Elite ABC Standard Kit Peroxidase) for 30 min at room temperature followed by AEC staining (Biologo, Kronshagen, Germany). For a negative control, the primary antibody was pre-incubated with a ∼100-fold molar excess of the immunizing peptide. Sections were mounted with Kaiser’s glycerol gelatine (Merck). For IF, we performed the same antigen retrieval and primary antibody incubation as outlined above, but used donkey anti-rabbit Cy3-coupled antibody (dianova, Hamburg, Germany, dilution 1∶200) as the secondary antibody. Sections were counterstained with DAPI (4',6-diamidino-2-phenylindole dihydrochloride, Life Technologies) and mounted with Fluorescent Mounting Medium (Dako). Pre-incubation control was performed as outlined above. In addition to the detection of SOAT, we performed IHC and IF with a specific marker for the Golgi apparatus, Golgin A2 (GOLGA2), which is also known as GM130 [36].

### Western Blot Experiments

For WB analysis of stably transfected SOAT-HEK293 cells, the cells were cultured to 100% confluence under standard conditions and pre-incubated with tetracycline (1 µg/ml, Carl Roth GmbH, Karlsruhe, Germany) to induce SOAT expression, as reported previously [29]. SOAT-HEK293 cells without tetracycline pre-treatment, as well as non-transfected HEK293 cells, were used as controls. For protein extraction, culture medium was removed and cells were washed with PBS. 400 µl ice-cold RIPA buffer (Sigma-Aldrich) mixed with protease inhibitor at a 1∶100 dilution (Thermo Fisher Scientific, Waltham, MA, USA) were added to the cells. After 15 min on ice, cells were detached and mechanically destroyed, and left on ice for further 10 min. Then, the lyzed cells were centrifuged for 15 min at 13,000 rpm and 4°C. The supernatant was obtained and used for determination of the protein content. Afterwards, samples were mixed with 4× Lämmli buffer containing 20% ß-mercaptoethanol and separated on a 10% SDS-PAGE over night at 50 V. The gel was blotted on a nitrocellulose membrane using semi-dry electroblotting and protein transfer was controlled by placing the nitrocellulose membrane in Ponceau S (Sigma-Aldrich) for 5 min. After multiple washing with Tris-buffered saline TBS-T (137 mM NaCl, 10 mM Tris-HCl, pH 8.0, 0.05% Tween-20), the membrane was blocked for 1 h at room temperature under agitation in TBS-T with 5% dried non-fat milk (blocking solution). After removal of the blocking solution, the nitrocellulose membrane was incubated with the primary antibody in blocking solution (1∶100 dilution) for 1 h at room temperature. After washing with TBS-T, the membrane was incubated with the peroxidase-conjugated goat IgG fraction to rabbit IgG secondary antibody (MP Biomedicals, Pioneer Place, Singapore), at a 1∶5,000 dilution together with the Roti Mark Western-HRP-conjugate (1∶5,000 dilution, Carl Roth) for 1 h at room temperature. After washing with TBS-T, the nitrocellulose membrane was incubated with Roti Lumin 1 and Roti Lumin 2 (Carl Roth) in a ratio of 1∶1 for 1 min and exposed to Amersham Hyperfilm ECL High Performance chemiluminescence film (GE Healthcare, LifeSciences, Piscataway, NJ, USA).

### Stably Transfected SOAT-, OATP6A1-, OSCP1-, and OATP1C1-HEK293 Cells

The recombinant human cell line T-REx-SOAT-HEK293 was established in 2007 and demonstrated the transport of sulfated steroid hormones [29]. Using the same method, here we generated stably transfected OATP6A1-, OSCP1-, and OATP1C1-HEK293 cell lines. Briefly, full length transcripts for OATP6A1 and OSCP1 were obtained by RT-PCR from human testis cDNA (BioChain) as a template. OATP1C1 was cloned from human brain cDNA (BioChain) because of its higher expression rate in this organ. The used primers are displayed in Table 3. Using the Phusion High Fidelity PCR Kit (Thermo Fisher Scientific) the PCR reactions were performed under the following thermocycling conditions: 1×98°C for 30 s, 10×(98°C for 10 s, 68°C (OATP6A1) or 62°C (OATP1C1, OSCP1) for 30 s, decreasing 0.5°C each cycle, and 72°C for 1 min) followed by 35×(98°C for 10 s, 63°C (OATP6A1) or 57°C (OATP1C1, OSCP1) for 30 s, 72°C for 1 min) and 72°C for 10 min. PCR products were visualized on a 1% agarose gel. The amplicons were excised from the gel and purified using Hi Yield PCR Clean-up+Gel-Extraction Kit (SLG, Gauting, Germany). In order to clone the full length transcript of each carrier via T/A cloning into the pcDNA5/FRT/TO TOPO or pcDNA5/FRT/V5-His TOPO vectors (LifeTechnologies), a 3′-deoxyadenosine-overhang was attached to the blunt ends of the carrier cDNAs by incubating the purified PCR-product with DyNAzyme II DNA Polymerase (Thermo Fisher Scientific) and dATPs for 10 min at 72°C. All clones were sequence verified according to the following GenBank Accession Numbers: NM_173488 for OATP6A1, NM_017435 for OATP1C1 and NM_145047 for OSCP1. The carrier-pcDNA5 constructs were co-transfected with the Flp recombinase expression vector pOG44 into Flp-In T-REx 293 host cells by lipofectamine 2000 (LifeTechnologies) transfection according to the manufactureŕs protocol. Stably transfected clones containing the gene of interest were selected by culturing in selective medium containing 150 µg/ml hygromycin and 50 µg/ml blasticidine (Carl Roth). After 14 to 16 days, single clones containing the full open reading frame of the respective carrier were selected and used for further experiments. Respective HEK293 cells were cultured in DMEM/F-12 medium (LifeTechnologies), supplemented with 10% FCS (Sigma-Aldrich), L-glutamine (4 mM), penicillin (100 units/ml), and streptomycin (100 µg/ml) at 37°C, 5% CO_2_, and 95% humidity.

**Table 3:** Primers used for full length amplification of the carrier open reading frames.

Target	RefSeq	Primer sequence (5′ → 3′)	amplicon (bp)
	(NCBI database)		
OATP6A1	NM_1743488.3	CAGGGTGAGCCATGTTCGTAG *for*	2186
		ACAATGATGATCCAGTTACAAGTCAG *rev*	
OATP1C1	NM_017435.4	ATAATGGACACTTCATCCAAAG *for*	2153
		AAGTGGAGGTTTCCTTGCCTG *rev*	
OSCP1	NM_145047.4	CTCGTTTCCAGCACCATGTC *for*	1166
		GGTCAGAACAGCTATAACTCA *rev*	

### Immunofluorescence Microscopy of Stably Transfected Cell Lines

For immunofluorescence microscopy, cells were seeded on poly-D-lysine coated glass coverslips in 24-well plates with a density of 1×10^5^ cells per well. SOAT expression was induced by tetracycline treatment (1 µg/ml). SOAT-HEK293 cells grown up in the absence of tetracycline were used as control as well as SOAT non-expressing cells (Flp-In-HEK293 cells) which were also maintained without tetracycline. After 72 h, cell medium was removed and cells were washed with PBS for 5 min. The following experimental steps were performed at room temperature. The cells were fixed with 2% paraformaldehyde in PBS for 15 min and subsequently permeabilized for 5 min in PBS buffer supplemented with 0.2% Triton X-100 and 20 mM glycine. After washing the cells with PBS for 5 min, cells were placed in blocking solution containing 20 mM glycine in PBS and 4% goat serum for 1 h. Afterwards, cells were incubated with the SOAT_311–377_ antibody at a dilution of 1∶100 in blocking solution for 1 h. Cells were washed three times with PBS and incubated with the Alexa Fluor 555-labeled goat anti-rabbit IgG secondary antibody (LifeTechnologies) at 1∶800 dilution in blocking solution for 1 h. After a final washing procedure, cells were covered with a DAPI/methanol solution containing 1 µg/ml DAPI and incubated for 1 minute. The cells were washed with methanol, air-dried and mounted on slides with ProLong Gold Antifade Reagent (LifeTechnologies). Fluorescent imaging was performed on a Leica DM5500B microscope (Leica, Bensheim, Germany). Captured images were analyzed with the Leica Fluorescence Workstation software LAS AF 6000.

### Transport Studies of Stably Transfected HEK293 Cells

All of the chemicals, unless otherwise stated, were obtained from Sigma-Aldrich. [^3^H]DHEAS and [^3^H]E1S were purchased from PerkinElmer Life Sciences (Waltham, MA, USA). For all transport studies, 24-well plates were coated with poly-D-lysine for better attachment of the cells. 1.25×10^5^ cells per well were seeded and cultured with standard medium for 72 hours containing tetracycline (1 µg/ml) to induce the carrier expression. Cells without tetracycline pre-treatment and cells without the gene of interest were used as control. OATP1C1-HEK293 cells were not pre-treated with tetracycline as they constitutively express OATP1C1. Transport studies with SOAT-HEK293 cells were performed in the presence and absence of sodium (control). In the sodium-free transport buffer, sodium chloride was replaced by equimolar concentrations of choline chloride. Before starting the transport studies, cells were washed three times with PBS. After washing, cells were pre-incubated with transport buffer (142.9 mM NaCl, 4.7 mM KCl, 1.2 mM MgSO_4_, 1.2 mM KH_2_PO_4_, 1.8 mM CaCl_2_, and 20 mM HEPES, adjusted to pH 7.4 with KOH, 37°C) for 15 min. Transport studies were performed by incubating cells with 250 µl transport buffer containing the radiolabeled or non-radiolabeled compounds for 10 min at 37°C. Uptake studies were stopped by removing the transport buffer and washing the cells 5 times with ice-cold PBS. Afterwards, cells were lyzed in 1 N NaOH with 0.1% SDS and the cell-associated radioactivity was measured by liquid scintillation counting as described before [29]. For LC-MS-MS analysis of the transportates, cells were lyzed with water by three freeze-thaw cycles and uptake of the non-radiolabeled compounds androstenediol-3-sulfate, β-estradiol-3-sulfate (both purchased from Steraloids Fountain Limited, Malta), estrone-3-sulfate, and DHEAS was measured as described by Galuska et al. 2012 [37]. The protein content was calculated using the BCA protein assay kit from Novagen (EMD Biosciences, Madison, WI, USA).

## Results

### Expression Pattern of SOAT, OATP6A1, OATP1C1 and OSCP1 in Human Tissues

**Figure 1:**
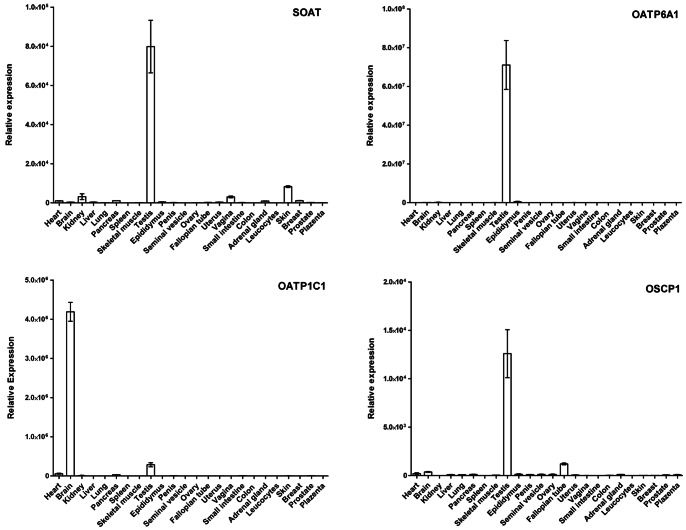
Expression pattern of SOAT, OATP6A1, OATP1C1 and OSCP1 in human tissues. Tissue expression of the indicated carriers was analyzed by quantitative real-time PCR. Relative expression depicted at the y-axis represents x-fold higher expression in the respective tissue compared to the tissue with the overall lowest expression among the tissue panel (set as calibrator). The values represent means ± SEM of triplicate determinations.

In order to systematically analyze the tissue mRNA expression levels of the carriers SOAT, OATP6A1, OATP1C1, and OSCP1 which previously showed high expression in the testis, we used comprehensive human cDNA panels. As shown in Fig. 1, SOAT was predominantly expressed in the testis and mRNA expression was also detected in skin, kidney, vagina, pancreas, adrenal gland and breast. Furthermore, OATP6A1 and OSCP1 were predominantly expressed in the testis with minor expression in the epididymis and fallopian tube, respectively. In contrast, OATP1C1 is predominantly expressed in the brain with further, but minor expression in the testis. Therefore, further expression analyses in the human testis focused on SOAT, OATP6A1 and OSCP1.

### Quantitative Expression Analysis in Testicular Biopsies

**Figure 2:**
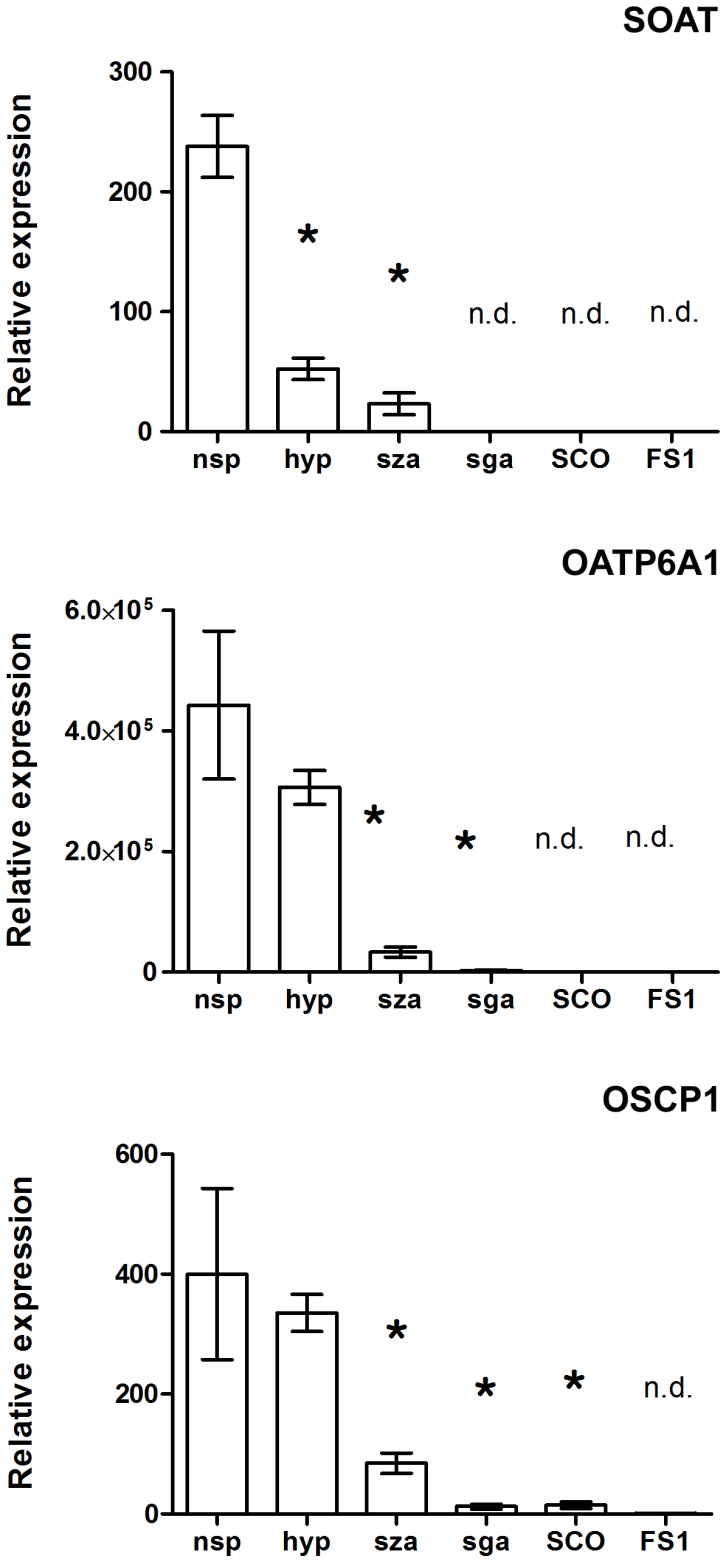
Quantitative real-time PCR analysis of the SOAT, OATP6A1 and OSCP1 expression in human testis biopsies with normal or impaired spermatogenesis. Expression analysis was performed with testis biopsies revealing normal spermatogenesis (nsp, n = 12), qualitatively intact but quantitatively reduced spermatogenesis (hypospermatogenesis, hyp, n = 5), arrest at the level of primary spermatocytes (sza, n = 13) or spermatogonia (sga, n = 5) as well as Sertoli cell only syndrome (SCO, n = 9). Furthermore, expression analysis was performed on human FS1 Sertoli cell cultures. *Significantly lower expression compared to nsp with p<0.001. Data represent mean ± SEM; n.d., expression of the respective carrier was not detected in any biopsy. In the case that only single biopsies from the probe collection showed no detectable expression, the C_T_ value in these samples was set to 40.

The data from the tissue cDNA panels were further verified in testes biopsies showing intact normal spermatogenesis (nsp, n = 12), which all showed very high expression levels for SOAT, OATP6A1 and OSCP1 (Fig. 2). Interestingly, in patients with hypospermatogenesis (hyp), which is characterized as a quantitatively reduced but qualitatively preserved spermatogenesis, SOAT expression was significantly lower compared to nsp. Expression levels of OATP6A1 and OSCP1 were not significantly reduced in hyp. However, for all three carriers we found significantly lower or even absent mRNA expression in severe disorders of spermatogenesis, represented by an arrest at the level of spermatocytes (sza) or spermatogonia (sga), or even by a total loss of germ cells (Sertoli cell only syndrome, SCO). Similar to SCO, none of these carriers were detected in cultured Sertoli cells (FS1), indicating all of them to be expressed in germ cells.

### Qualitative Expression Analysis following LACP of Testes Biopsies

**Figure 3:**
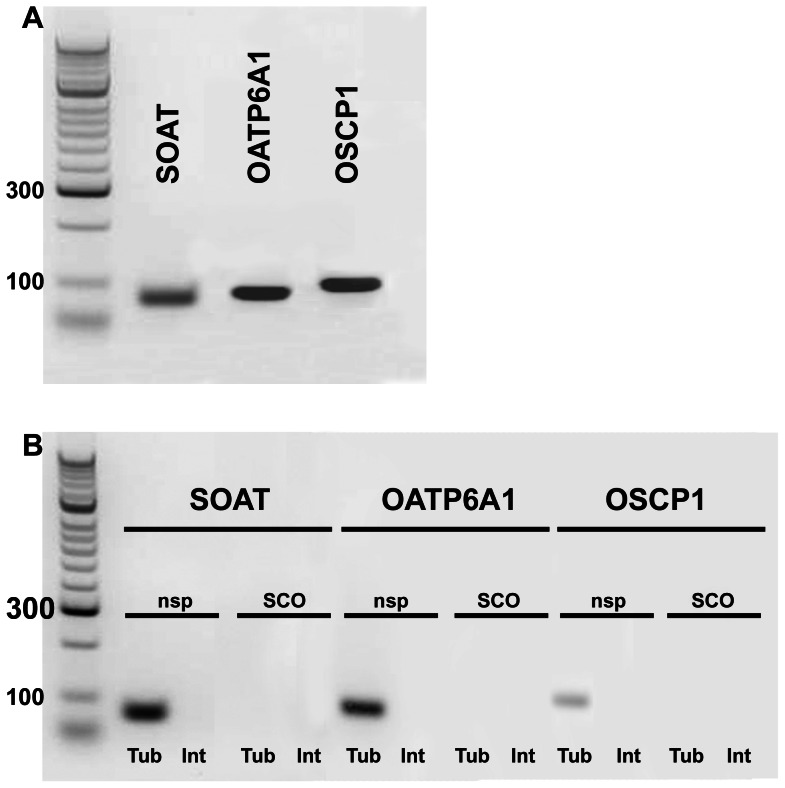
Qualitative mRNA expression analysis of SOAT, OATP6A1 and OSCP1 in seminiferous tubules (Tub) and interstitial tissue (Int) of human testes biopsies showing normal or impaired spermatogenesis following LACP. (**A**) Expression of all three carriers was detected in testis homogenate showing normal spermatogenesis. (**B**) SOAT, OATP6A1, and OSCP1 were only detected in seminiferous tubules from testis biopsies showing nsp, but not in the tubules from patients with SCO. No carrier mRNA was detected in interstitial tissue of the biopsies regardless their spermatogenic status. M, marker.

In order to verify this assumption, we used qualitative RT-PCR analysis following LACP of testes biopsies from patients showing nsp or SCO. In this approach, interstitial tissue (encompassing Leydig cells, blood vessels and connective tissue; indicated by “Int”) was separated from seminiferous tubules (containing peritubular cells, Sertoli cells and germ cells, indicated by “Tub”). All three carriers were only detected in the seminiferous tubules of patients with nsp, but not in tubules from SCO biopsies as well as in interstitial tissue. Therefore, germ cell specific expression of SOAT, OATP6A1 and OSCP1 was confirmed (Fig. 3).

### Cellular Localization of SOAT in the Human Testis

**Figure 4:**
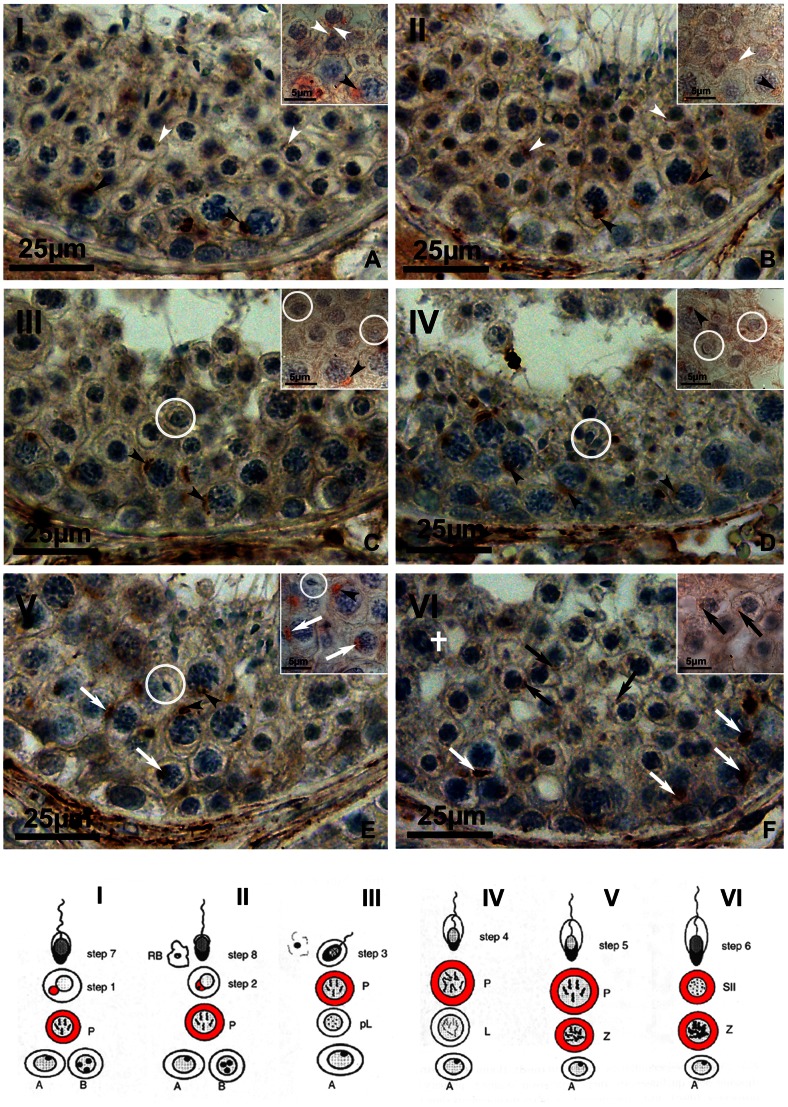
Immunohistological localization of SOAT in normal spermatogenesis after stage-dependent analysis. IHC was performed using the Soat_329–344_ antibody with subsequent AEC staining and hematoxylin counterstain. The larger pictures show a whole segment of the seminiferous epithelium in different stages (I–VI) of spermatogenesis (primary magnification ×40), whereas insets show a detail of the respective stage (primary magnification ×100 oil). SOAT expression is also schematically indicated by red labeling on a drawing of spermatogenic stages (modified from [34]). (**A**) Within stage I of spermatogenesis, the SOAT protein was detected in primary pachytene spermatocytes (P, black arrowheads) and round spermatids (step 1, white arrowheads). (**B**) SOAT immunoreactivity was detected in stage II of spermatogenesis within primary pachytene spermatocytes (P, black arrowheads) as well as in round spermatids (step 2, white arrowheads). (**C**) In stage III, only primary pachytene spermatocytes were stained with the Soat_329–344_ antibody (P, black arrowheads), whereas round spermatids were negative (step 3, white circles). (**D**) Within stage IV of spermatogenesis, a similar staining pattern was detected, showing SOAT protein in primary pachytene spermatocytes (P, black arrowheads), but not in round spermatids (step 4, white circles). Note the newly formed acrosomal cap in step 4 spermatids. (**E**) In stage V, primary zygotene spermatocytes (Z, white arrows) as well as pachytene spermatocytes (P, black arrowheads) were stained. Elongating spermatids, showing a distinct acrosomal cap, were not stained (step 5, white circles). (**F**) Stage VI is characterized by the first meiotic cleavage (white cross). Positive staining for SOAT protein was detected in primary zygotene spermatocytes (Z, white arrows) as well as in secondary spermatocytes (SII, black arrows), which can be hardly distinguished from round spermatids.

**Figure 5:**
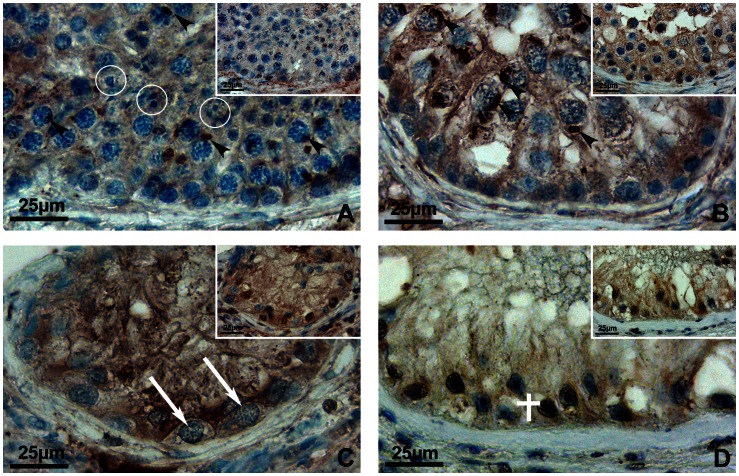
Localization of SOAT in impaired spermatogenesis by IHC. IHC was performed using the Soat_329–344_ antibody with subsequent AEC staining and hematoxylin counterstain. For negative control, primary antibody was incubated with a 100-fold molar excess of immunizing peptide (pre-incubation control, insets). Primary magnification ×40. (**A**) In a seminiferous tubule showing hypospermatogenesis, i.e. a quantitatively reduced but qualitatively preserved spermatogenesis, SOAT specific staining was detected in primary pachytene spermatocytes (black arrowheads), but not in round or elongated spermatids (white circles). (**B**) In a spermatogenic arrest at the level of primary spermatocytes, only these cells are present, whereas the development of spermatids is missing. In this seminiferous tubule, primary spermatocytes were stained for SOAT (black arrowheads). (**C**) In an arrest of spermatogenesis at the level of spermatogonia, no primary spermatocytes are present, but spermatogonia (white arrows) are left. No specific staining for SOAT was detected in these tubules. (**D**) In SCO, no germ cells are left; only Sertoli cells (white cross) are visible and show a faint unspecific cytoplasmic staining with the Soat_329–344_ antibody, which was not abolished in the pre-incubation control.

For immunolocalization of the carrier proteins in the human testis several antibodies were considered and tested, but most of them were not applicable for immunohistochemistry or showed no specific staining pattern. In the case of SOAT, we obtained two antibodies from custom immunization (Eurogentec, Belgium). The first was derived from the complete C-terminal amino acid sequence of the human SOAT protein (referred to as SOAT_311–377_) and the second was generated from the 16 C-terminal amino acids of the mouse Soat protein (referred to as Soat_329–344_). However, only Soat_329–344_ was applicable for immunohistochemistry (Fig. 4 and 5) and only SOAT_311–377_ was suitable for Western blot analysis.

**Figure 6:**
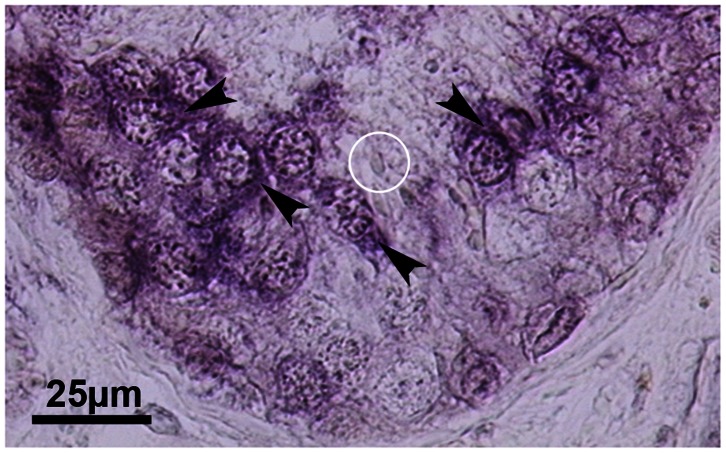
Detection of SOAT mRNA by ISH. Expression analysis of SOAT mRNA in the human testis was also performed using *in situ* hybridization on human testis biopsies showing normal spermatogenesis. Even at the mRNA level, SOAT expression was detected in pachytene primary spermatocytes (black arrowhead) within the seminiferous tubules at late stage II of spermatogenesis. Spermatids were not stained (white circle). Incubation with sense probe showed no staining signal. NBT-BCIP staining, hematoxylin counterstain. Primary magnification ×40.

**Figure 7:**
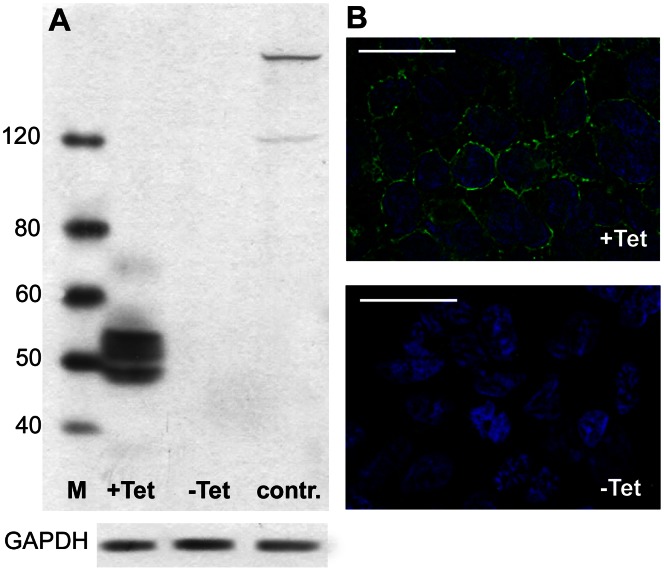
SOAT expression in stably transfected SOAT-HEK293 cells. In the SOAT-HEK293 cells SOAT expression was induced by pre-treatment with tetracycline (+Tet). Control cells were untreated with tetracycline (−Tet) or represent non-transfected HEK293 cells (contr.). (**A**) Cell lysates were processed for WB analysis with the SOAT_311–377_ antibody and revealed an apparent molecular weight of 49–55 kDa, likely representing different glycosylation states of the SOAT protein. (**B**) SOAT expression was directed to the plasma membrane of HEK293 cells by immunofluorescence analysis with the SOAT_311–377_ antibody (green fluorescence). Nuclear staining with DAPI (blue fluorescence). Scale bar: 25 µm.

**Figure 8:**
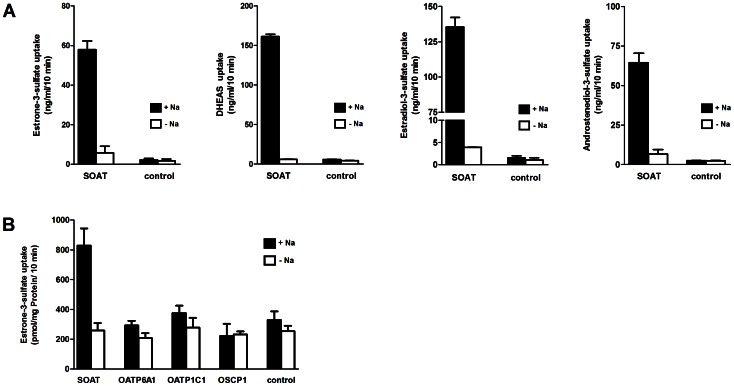
Transport studies with sulfated steroid hormones. (**A**) SOAT-HEK293 were incubated with 10 µM non-radiolabeled estrone-3-sulfate, DHEAS, β-estradiol-3-sulfate, and androstenediol-3-sulfate in the presence (black bars) or absence (open bars) of sodium over 10 min at 37°C. Cells lysates were analyzed by LC-MS-MS in order to determine the absolute cell-associated amount of the steroid sulfate molecules in their intact forms. (**B**) Transport studies with 10 µM [^3^H]estrone-3-sulfate on stably transfected SOAT-, OATP6A1-, OATP1C1-, and OSCP1-HEK293 cells in the presence and absence of sodium. Non-transfected HEK293 cells served as a control. In contrast to SOAT, OATP6A1, OATP1C1, and OSCP1 showed no significant transport function for E1S.

Therefore, the Soat_329–344_ affinity-purified rabbit antiserum was used to localize SOAT in the human testis. As shown in Fig. 4 and Fig. S1, the immunoreactivity was clearly directed against various germ cell stages. Staining was found in zygotene primary spermatocytes of stage V, pachytene spermatocytes of all stages (I–V), secondary spermatocytes of stage VI and round spermatids (step 1 and step 2) in stages I and II. Round spermatids of stage III were not stained. Most prominent was the staining of primary spermatocytes, which are germ cells within the first meiotic division. They are characterized by their cellular and nuclear morphology, showing distinct chromosomal structures due to condensation prior to first meiotic cleavage. Here, an ovoid-shaped structure close to the nucleus was stained, likely representing the Golgi compartment, whereas no clear staining was detected in the plasma membrane. This was also shown by *in situ* hybridization (Fig. 6). In stably SOAT-transfected HEK293 cells, SOAT protein was detected via Western blot (Fig. 7A) and immunofluorescence. Contradictory to the localization in germ cells, we detected a clear plasma membrane-derived staining pattern in stably SOAT-transfected HEK293 cells (see Fig. 7B) and the functional characteristics of SOAT as a sodium-dependent steroid sulfate uptake carrier in the plasma membrane (see Fig. 8). However, this staining pattern of SOAT was regarded as specific as it was clearly abolished by pre-incubation of the antibody with the immunizing peptide as well as omission of the primary antibody. In order to further analyze the sub-cellular localization of SOAT in primary spermatocytes IHC and IF of consecutive human testis sections were performed with the Soat_329–344_ antibody as well as an antibody against the Golgi apparatus protein Golgin A2. As shown in Fig. S2, SOAT and Golgin A2 showed identical expression patterns within primary spermatocytes. Apart from germ cells, a diffuse staining of the testis interstitial compartment was visible with the Soat_329–344_ antibody. However, as this staining remained nearly unchanged after pre-incubation of the antibody with the immunizing peptide, it was regarded as unspecific staining (insets of Fig. 5).

In impaired spermatogenesis, as hypospermatogenesis, spermatogenic arrest and SCO, no staging of spermatogenesis is possible. In these sections, we detected the SOAT protein in primary pachytene spermatocytes in case these cells were present (Fig. 5). In SCO tubules, an unspecific staining of Sertoli cell cytoplasm was visible, that did not disappear after pre-incubation of the antibody with the immunizing peptide and, therefore, was regarded as unspecific staining (Fig. 5D). Apart from IHC, we performed *in situ* hybridization experiments using anti-sense cRNA probes for detection of the cellular SOAT mRNA expression in the human testis. As shown in Fig. 6, SOAT mRNA was detected in pachytene spermatocytes, whereas Sertoli cells, peritubular cells, and the interstitial compartment were not stained, clearly supporting the IHC localization data.

### Transport of Steroid Sulfates across the Plasma Membrane by SOAT

A further aim of the present study was to verify whether SOAT indeed can transport sulfated steroid hormones without hydrolysis from the extracellular compartment into the cytosol. Therefore, a novel LC-MS-MS procedure was used that is capable of profiling intact steroid sulfates from cell lysates. Transport assay were performed in stably transfected SOAT-HEK293 cells in which SOAT expression was induced by tetracycline treatment (+Tet), whereas non-treated SOAT-HEK293 cells (−Tet) served as a control. The latter did not express the SOAT protein, as demonstrated by Western blot and immunofluorescence analysis with the SOAT_311–377_ antiserum (Fig. 7). As shown in Fig. 8A, we demonstrated for the first time sodium-dependent transport of β-estradiol-3-sulfate (E2S) and androstenediol-3-sulfate by SOAT-HEK293 cells. Furthermore, we verified the sodium-dependent transport of estrone-3-sulfate and DHEAS by SOAT that was previously shown by using the respective radiolabeled compounds. However, apart from SOAT, we could not detect transport activity for human OATP6A1, OATP1C1, or OSCP1 for any of the steroid sulfates and also not for taurocholic acid (a common probe substrate for most OATP carriers) in respective OATP6A1, OATP1C1, and OSCP1 stably-transfected HEK293 cells (Fig. 8B). Even after expression of human OATP6A1, OATP1C1 and OSCP1 in *Xenopus laevis* oocytes as a second expression system, no transport activity could be detected for steroid sulfates (data not shown), indicating that SOAT seems to be the only relevant steroid sulfate uptake carrier in the testis among the carriers analyzed.

## Discussion

Sulfated steroids are present in the blood circulation at quite high physiological concentrations, for example up to 10 µM in the case of DHEAS [38]. Apart from being excreted via bile and urine, these compounds can also be de-sulfated into biologically active steroid hormones and may therefore contribute to the overall regulation of reproductive processes [39]. In the case of PREGS and DHEAS, which represent the most abundant sulfated steroids in the human testis, these compounds, together with androstenediol-3-sulfate were shown to be metabolized to testosterone in the human testis, at least in tissue homogenates [8], [16], [40]. However, *in vivo* this would require a transport process for these hydrophilic anionic compounds of generally low membrane permeability across membrane barriers in the testis. Therefore, in the present study we focused on the localization and characterization of candidate steroid sulfate carriers in the human testis and showed that SOAT, OATP6A1 and OSCP1 reveal very high and predominant mRNA expression levels in this organ. However, as only SOAT showed significant transport activity for different steroid sulfate molecules, including PREGS, DHEAS, androstenediol-3-sulfate, E1S and E2S, we primarily aimed to localize this carrier in the human testis.

Interestingly, we found SOAT specifically expressed in primary (zygotene and pachytene) as well as secondary spermatocytes and in round spermatids (step 1 and step 2) within normal spermatogenesis with an immunostaining of ovoid-shaped structures close to the nucleus. As these structures were also immunoreactive for Golgin A2, a well-established marker of the Golgi apparatus [36], SOAT expression in germ cells is most likely directed to the Golgi compartment. As all previous work on SOAT [27], [29] and also the data from the present study localized SOAT in the plasma membrane and showed the sodium-dependent uptake of steroid sulfates across the plasma membrane, at least in transfected HEK293 cells and *Xenopus laevis* oocytes, we consider this expression pattern to be an intermediate sorting state of the protein for its further trafficking to the plasma membrane at later stages of germ cell development. Here, the protein may then be largely distributed over the plasma membrane so that immunostaining is no longer detectable with the IHC parameters used. However, we cannot exclude that the primary target compartment of SOAT is represented by the immunostained complex within germ cells. In every case, this expression site of SOAT in the human testis can be regarded as specific and is largely supported by ISH analysis and RT-PCR following LACP of seminiferous tubules with nsp or SCO. In impaired spermatogenesis, represented by hypospermatogenesis, spermatogenic arrests and SCO, SOAT protein was also detected in primary spermatocytes in cases these cells were present. In biopsies with SCO, the Sertoli cell cytoplasm was additionally stained with the Soat_329–344_ antibody. However, as this staining remained in the pre-incubation control, it was regarded as unspecific. This was further confirmed by quantitative RT-PCR analysis of cultured FS1 Sertoli cells, in which SOAT mRNA expression was not detectable.

Apart from the localization of SOAT in the human testis, we wanted to clarify whether SOAT indeed can transport sulfated steroid hormones without hydrolysis from the extracellular compartment into the cytosol. It has to be emphasized that most of the classical transport assays for solute carriers are performed with radiolabeled substrates by scintillation counting of the cell lysate after a certain time of incubation with the test compound from the extracellular site [29]. This procedure can cause two potential problems: (I) the availability of radiolabeled potential substrates is restricted, so that identification of novel substrates may be missed, and (II) it is difficult to demonstrate transport of the intact substrate from the extracellular compartment into the cytosol, because the radiolabel simply serves as a surrogate for detection of the entire molecule. To overcome these uncertainties for the steroid sulfate transport by SOAT, we used a LC-MS-MS procedure by which different sulfated steroid hormones can be detected from cell lysates in their intact forms [37]. LC-MS-MS currently presents the technique of choice for steroid sulfate detection, because mass spectrometry allows for the highest specificity in steroid analysis, and soft ionization enables determination of the intact steroid sulfate. Using this technique, we were able to expand the substrate spectrum of SOAT by androstenediol-3-sulfate and E2S using non-radiolabeled compounds. Furthermore, we could verify that the previously identified SOAT substrates DHEAS and E1S are transported in the SOAT-HEK293 cells from the extracellular into the intracellular compartment in their intact form without any modification at the molecule. Therefore, cellular import of sulfated steroid hormones seems to be the general transport function of SOAT.

However, the physiological role of steroid sulfate transport by SOAT in spermatocytes and spermatids remains largely unclear. In particular, DHEAS and PREGS are abundantly found in the human testis and are known to be precursors of testosterone [8], [41], so that transport of these compounds within the testis may play a role in the initiation and maintenance of spermatogenesis. However, germ cells do not express the androgen receptor, suggesting that androgens can only affect these cells in a non-genomic way [10], [42]. As germ cells are known to express estrogen receptors [6], [43], [44], the transport of E1S and E2S could be a relevant function of SOAT in spermatocytes. On this background it is interesting to note that SOAT expression was very low or even absent in severe disorders of spermatogenesis, represented by an arrest at the level of spermatocytes or spermatogonia, and also by SCO. These pathologies result in non-obstructive azoospermia and, therefore, infertility in men [34]. However, from these data, no causal relationship can be concluded between low SOAT expression and severe spermatogenic impairment. In patients with hypospermatogenesis characterized by qualitatively preserved, but quantitatively reduced spermatogenesis, SOAT expression was significantly lower compared to normal spermatogenesis. As a significant reduction of SOAT expression may be associated with a disturbed transport of sulfated steroids, the local supply of androgens and estrogens may be disturbed. One possible way to evaluate this hypothesis would be the analysis of spermatogenesis in the testis of Slc10a6 knockout mice lacking any Soat expression. Furthermore, among the known ≥33 non-synonymous single nucleotide polymorphisms (SNPs) in the human SLC10A6 gene, functionally relevant SNPs might be more represented in patients with hypospermatogenesis or other spermatogenic impairments.

Apart from SOAT, OATP6A1 and OSCP1 also showed predominant expression in the human testis. This is in agreement with previous studies that were performed on mice, rats and humans [23], [24], [29], [32]. However, by using stably transfected OATP6A1-HEK293 and OSCP1-HEK293 cells, and even *Xenopus laevis* oocytes as second expression system, we could not show any significant transport activity for DHEAS and E1S. OATP6A1 was cloned by Suzuki et al. 2003 [23] from rats and humans, and was localized in Sertoli cells, spermatogonia, and Leydig cells of the rat testis by ISH. Functional transport measurements were only performed with the rat carrier and revealed transport activity for taurocholic acid and T_4_, as well as low transport rates for DHEAS [23], which is in contrast to the human OATP6A1 carrier. OSCP1 was cloned from human, rat, and mouse, and showed transport activity for various kinds of organic solutes including DHEAS and E1S with very low transport rates [24], [32], [45]. The mouse Oscp1 protein was localized to leptotene spermatocytes at stage IX until step 15 spermatids in the mouse testis. However, Oscp1 was clearly found intracellularly [30]; therefore, it is still controversial whether OSCP1 acts as a membrane uptake carrier at all. As both carriers were transport negative for DHEAS and E1S in our expression models, their cellular localization in the human testis was not analyzed further.

With the present study, we were able to limit the localization of SOAT to germ cells. Nevertheless, how the sulfated steroids reach the germ cell stages still remains an open question. SOAT positive primary and secondary spermatocytes as well as round spermatids are located within the adluminal compartment of the seminiferous epithelium, built by Sertoli cells. The blood-testis barrier (first described by Bergmann et al. 1989 in the human [46]) protects germ cells from endogenous and exogenous substances and there is increasing interest regarding active transporter (ATP-binding cassettes, ABC) expression in Sertoli cells. Sulfated steroids have to pass through Sertoli cells to reach the germ cells, which could be mediated by ABC transporters like the multidrug-resistance proteins MRP1 and MRP4 as well as breast cancer resistance protein (BCRP). These have already been detected in human Sertoli cell lines and are thought to play a pivotal role in drug transport in the testis (for review see [47]).

## Supporting Information

Figure S1 **Overview on SOAT expression in different germ cell stages in the human testis.** IHC was performed using the Soat_329–344_ antibody with subsequent AEC staining and hematoxylin counterstain. For negative control, primary antibody was incubated with a 100-fold molar excess of immunizing peptide (pre-incubation control, inset). Primary magnification ×20. Within the depicted seminiferous tubule different spermatogenic stages (stages I, III and IV) as well as mitotic divisions (mit.) are present. The following cell types and structures can be assigned: **a,** spermatogonia; **b,** primary pachytene spermatocytes; **c,** early round spermatids; **d,** elongating spermatids; **e,** elongated spermatids prior to sperm release; **f,** Sertoli cell nucleus; **g,** interstitial Leydig cells; **h,** peritubular myoid cells. Specific SOAT expression can be detected in the present seminiferous tubule in pachytene spermatocytes of all stages as well as in round spermatids (step 1) in stage I. Round spermatids of stage III were not stained.
(TIF)

Figure S2 **Immunofluorescence and immunohistochemistry of consecutive sections of the human testis detecting SOAT and Golgi marker protein Golgin A2.** IHC and IF analyses were performed with the Soat_329–344_ (**A, C**) and Golgin A2 (**B, D**) antibodies in consecutive sections of the human testis. IHC was performed using AEC staining and hematoxylin counterstain. For IF nuclei were counterstained with DAPI (blue fluorescence). Negative controls were performed by pre-incubation of the Soat_329–344_ antibody with the immunizing peptide (insets in A, C) or by omitting the primary antibody (insets in B, D). Primary magnification ×40. (**A, B**) IF revealed specific staining of an ovoid-shaped structure close to the nucleus of primary spermatocytes (white arrowhead) with both antibodies (red fluorescence), representing the Golgi compartment. Notice unstained peritubular myoid cells (white circle). (**C, D**) The same staining pattern was observed using IHC, where SOAT and Golgin A2 showed identical expression patterns within primary spermatocytes (black arrowheads) in testis tissue sections showing normal spermatogenesis of stage III.
(TIF)
