# Supplementary figures and images for: Membrane Transporters for Sulfated Steroids in the Human Testis - Cellular Localization, Expression Pattern and Functional Analysis
Source: PLoS One. 2013 May 8;8(5):e62638. doi: 10.1371/journal.pone.0062638 (PMC3648580; doi:10.1371/journal.pone.0062638)

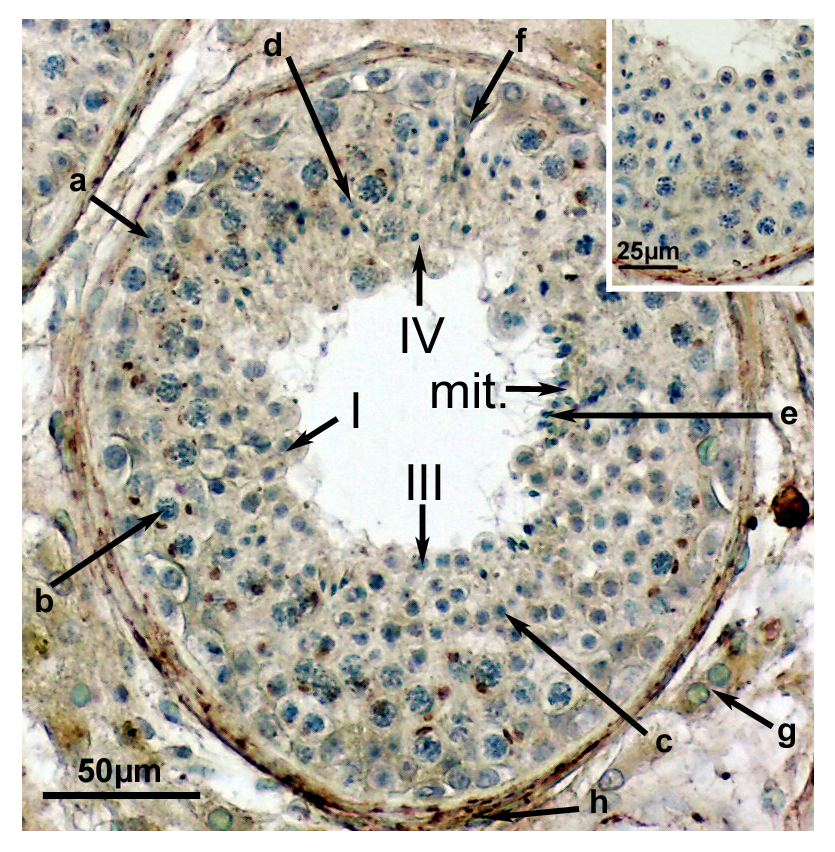

Supplement: Figure S1 — Overview on SOAT expression in different germ cell stages in the human testis. IHC was performed using the Soat329–344 antibody with subsequent AEC staining and hematoxylin counterstain. For negative control, primary antibody was incubated with a 100-fold molar excess of immunizing peptide (pre-incubation control, inset). Primary magnification ×20. Within the depicted seminiferous tubule different spermatogenic stages (stages I, III and IV) as well as mitotic divisions (mit.) are present. The following cell types and structures can be assigned: a, spermatogonia; b, primary pachytene spermatocytes; c, early round spermatids; d, elongating spermatids; e, elongated spermatids prior to sperm release; f, Sertoli cell nucleus; g, interstitial Leydig cells; h, peritubular myoid cells. Specific SOAT expression can be detected in the present seminiferous tubule in pachytene spermatocytes of all stages as well as in round spermatids (step 1) in stage I. Round spermatids of stage III were not stained. (TIF) [file pone.0062638.s001.tif]

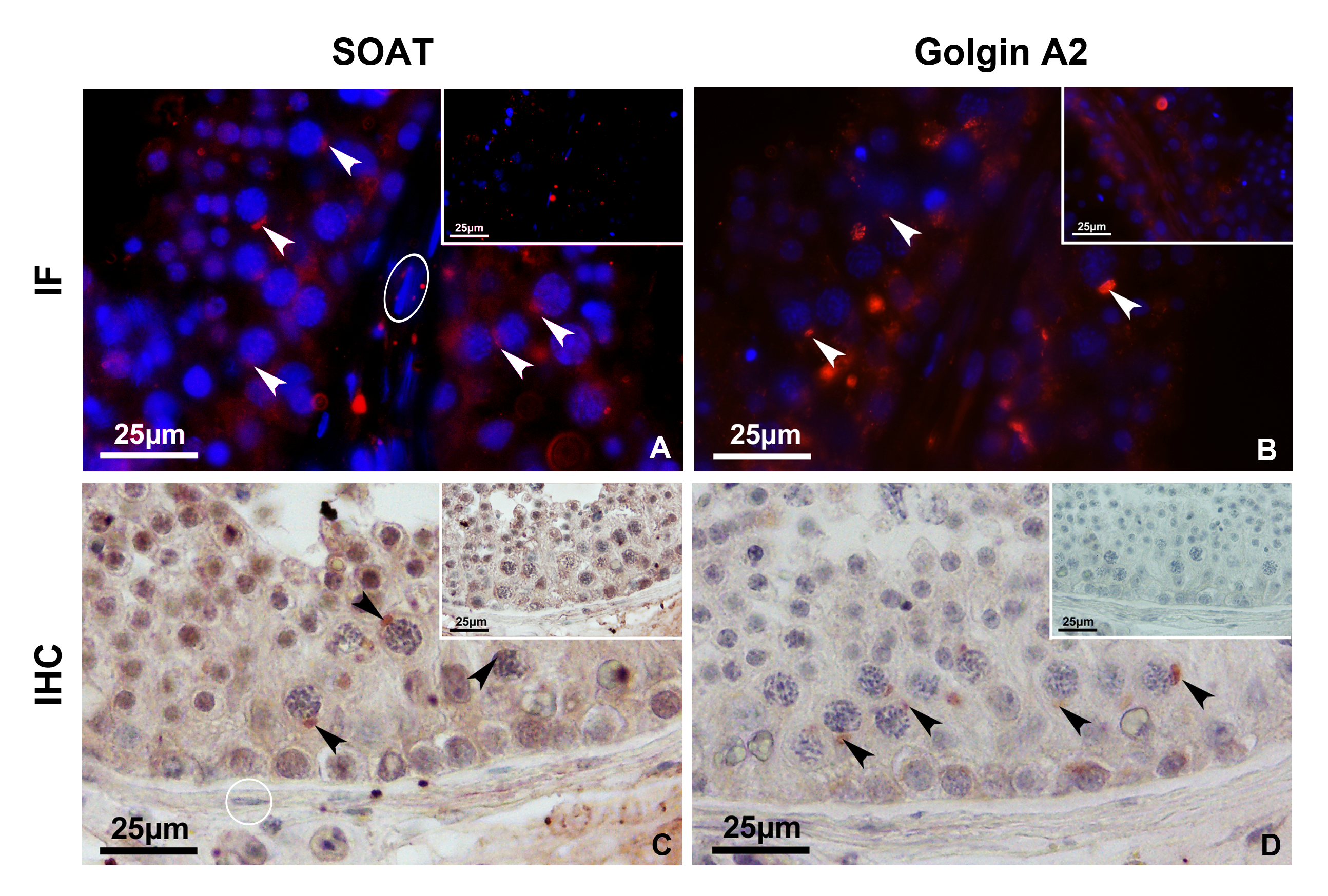

Supplement: Figure S2 — Immunofluorescence and immunohistochemistry of consecutive sections of the human testis detecting SOAT and Golgi marker protein Golgin A2. IHC and IF analyses were performed with the Soat329–344 (A, C) and Golgin A2 (B, D) antibodies in consecutive sections of the human testis. IHC was performed using AEC staining and hematoxylin counterstain. For IF nuclei were counterstained with DAPI (blue fluorescence). Negative controls were performed by pre-incubation of the Soat329–344 antibody with the immunizing peptide (insets in A, C) or by omitting the primary antibody (insets in B, D). Primary magnification ×40. (A, B) IF revealed specific staining of an ovoid-shaped structure close to the nucleus of primary spermatocytes (white arrowhead) with both antibodies (red fluorescence), representing the Golgi compartment. Notice unstained peritubular myoid cells (white circle). (C, D) The same staining pattern was observed using IHC, where SOAT and Golgin A2 showed identical expression patterns within primary spermatocytes (black arrowheads) in testis tissue sections showing normal spermatogenesis of stage III. (TIF) [file pone.0062638.s002.tif]
